# Investigation of molecular mechanisms underlying tetracycline resistance in thermophilic *Campylobacter* spp. suggests that previous reports of *tet*(A)-mediated resistance in these bacteria are premature

**DOI:** 10.1186/s13099-019-0338-1

**Published:** 2019-11-09

**Authors:** Caoimhe Lynch, Kayleigh Hawkins, Helen Lynch, John Egan, Declan Bolton, Aidan Coffey, Brigid Lucey

**Affiliations:** 1 Department of Biological Sciences, Cork Institute of Technology, Rossa Avenue, Bishopstown, Cork, Ireland; 2 NRL Campylobacter, Backweston Laboratory Complex, Young’s Cross, Celbridge, Co. Kildare Ireland; 3 University College Dublin, Belfield, Dublin 4, Ireland; 4 Teagasc Food Research Centre, Ashtown, Dublin, Ireland

**Keywords:** *Campylobacter*, Tetracycline, *tet*(A), *tet*(O), Antibiotic resistance, Antimicrobial susceptibility, Antibiotic resistance mechanisms, Mobile genetic elements, Plasmids

## Abstract

The true prevalence of *tet*(A), which codes for a tetracycline efflux pump, in thermophilic *Camplyobacter* spp. requires clarification after reports emerged in Iran (2014) and Kenya (2016) of the novel detection of *tet*(A) in *Campylobacter*. During our investigation of antibiotic resistance mechanisms in a sample of Irish thermophilic *Campylobacter* broiler isolates, it was determined that 100% of tetracycline-resistant isolates (n = 119) harboured *tet*(O). Accessory tetracycline-resistance mechanisms were considered as tetracycline minimum inhibitory concentrations ranged from 4 to ≥ 64 mg/L. Primers previously reported for the detection of *tet*(A) in *Campylobacter* failed to produce an amplicon using a positive control strain (*Escherichia coli* K12 SK1592 containing the pBR322 plasmid) and a selection of *Campylobacter* isolates. Accordingly, we designed new *tet*(A)-targeting primers on SnapGene2.3.2 that successfully generated a 407 bp product from the positive control strain only. Further in silico analysis using BLASTn and SnapGene2.3.2 revealed that previously reported *Campylobacter tet*(A) sequences deposited on GenBank shared 100% homology with *Campylobacter tet*(O). We postulate that this gave rise to the erroneous report of a high *tet(A)* prevalence among a pool of Kenyan broiler *Campylobacter* isolates that were tested using primers designed based on these apparent *tet*(A) sequences. In conclusion, further work would be required to determine whether the homology between *tet*(A) potentially present in *Campylobacter* and known *tet*(A) genes would be sufficient to allow amplification using the primers designed in our study. Finally, the existence of *tet*(A) in thermophilic *Campylobacter* spp. remains to be demonstrated.

## Main text

We read with interest an article reporting the novel detection of *tet*(A) among thermophilic *Campylobacter* spp. poultry isolates in Iran [1] and the subsequent detection of *tet*(A) among a pool of *Campylobacter* spp. chicken isolates in Kenya [2]. It is a timely reminder of emerging antibiotic resistance associated with the mobilisation of genes from other bacterial genera. However, we believe that it remains to be determined whether *tet*(A) exists among thermophilic *Campylobacter* spp.

Tetracycline-containing therapeutics are the most commonly administered antimicrobial in poultry production and animal husbandry in Ireland, used for the treatment of enteric, respiratory and dermal infections [3, 4]. Tetracycline resistance in *Campylobacter* spp. is usually mediated by a ribosomal protection protein Tet(O), which confers resistance by preventing tetracycline ribosomal binding, thus abolishing the inhibitory effect of the antibiotic by preventing bacterial protein synthesis via association of aminoacyl tRNA with the bacterial ribosome [3, 5].

In our study, during the investigation of antibiotic resistance mechanisms among a sample of 350 Irish broiler *Campylobacter* spp. isolates, we were especially interested in tetracycline resistance genes, as resistance to tetracycline was most prevalent (34%) by phenotypic sensitivity testing (Unpublished 2019). Tetracycline-resistant isolates were preliminarily screened for the presence of *tet*(O), using the method described by Aminov et al. [6] and it was determined that 100% of tetracycline-resistant isolates harboured the *tet*(O) gene. However, accessory tetracycline-resistance mechanisms were considered as minimum inhibitory concentrations ranged from 4 to ≥ 64 mg/L. Moreover, the mobilisation of tetracycline-resistant determinants is associated with the presence of *tet* genes on plasmids [3]. Hence, the *tet*(A) gene, which codes for an efflux protein and has been reported to co-exist with *tet*(O) in *Campylobacter*, was considered as part of the investigation [1, 2]. However, we seek clarification about the results published in the referenced articles [1, 2], and the true prevalence of *tet*(A) among thermophilic *Campylobacter* spp.

We tested the *tet*(A) primers described by Abdi-Hachesoo et al. [1] (Table 1) but they failed to produce an amplicon using the positive control strain *Escherichia coli* K12 SK1592 containing the pBR322 plasmid (DSM 3879). In addition, a selection of tetracycline-resistant thermophilic *Camplyobacter* spp. isolates also failed to generate an amplicon. New *tet*(A)-targeting primers were thus designed on SnapGene2.3.2, based on homologous regions of the *tet*(A) gene from the pBR322 plasmid (GenBank J01749.1) and from the *Pseudomonas putida* strain Fars110 (GenBank JN937120.1) (Table 1)—the latter strain having been reported by Abdi-Hachesoo et al. [1] as a *tet*(A) positive control. A 407 bp product was successfully amplified using the new primers (Tet(A)-Camp-F and Tet(A)-Camp-R) with the *E. coli* K12 positive control strain but none of the tetracycline-resistant thermophilic *Camplyobacter* spp. isolates generated a product.

**Table 1:** Primer used for the detection of *tet*(O) and *tet*(A)

Primer	Sequence (5′–3′)	Amplicon size (bp)	Annealing temperature (°C)	References
TetO-FW	ACGGARAGTTTATTGTATACC	171	52	[6]
TetO-RV	TGGCGTATCTATAATGTTGAC			
Tet(A)-F	GTGAAACCCAACATACCCC	888	50	[1]
Tet(A)-R	GAAGGCAAGCAGGATGTAG			[7]
Tet(A)-Camp-F	ATCGTGGCCGGCATCACCGG	407	54	This study
Tet(A)-Camp-R	TCCTCGCCGAAAATGACCC			

With reference to the methods used in our study, DNA was extracted using PureLink™ Genomic DNA Mini Kit (Invitrogen, CA, USA). PCR mixtures (50 µL) contained 2.5U Amplitaq™ DNA polymerase (Applied Biosystems, CA, USA), 1× buffer I (Applied Biosystems, CA, USA), 2.5 mM magnesium chloride, 0.2 mM of each dNTP (Sigma Aldrich, MO, USA), 200 µM forward and reverse primer (Table 1) and 1 µL of genomic DNA (between 50 and 100 ng/µL starting concentration). The PCR cycling conditions were: 95 °C for 2 min, 35 cycles of 94 °C for 30 s, annealing temperatures as described in Table 1 for 30 s, 72 °C for 1 min and final extension at 72 °C for 5 min. Amplified *tet*(O) and *tet*(A) products were resolved by electrophoresis in a 2% and a 1.5% agarose gel, respectively. All primers used are listed in Table 1.

The failure of the *tet*(A) primers listed by Abdi-Hachesoo et al. [1] to produce an amplicon with the same *E. coli* (DSM 3879 positive control strain), under less stringent conditions, prompted further investigation within our study. In the Abdi-Hachesoo et al. [1] publication, the original *tet*(A) primer (Tet(A)-F and Tet(A)-R) reference is not listed in their bibliography, although these primers were previously reported by Maynard et al. [7] for the detection of *tet*(A) among Canadian swine *E. coli* isolates (Table 1) [1, 7].

We scanned the *C. coli* and *C. jejuni tet*(A) sequences, Shiraz3 and Shiraz4 (GenBank accession numbers JX891463.1 and JX891464.1, respectively) deposited in GenBank in the Abdi-Hachesoo et al. [1] publication against all *Campylobacteraceae* (taxid 72294) sequences using BLASTn. Multiple *tet*(O) sequences were returned with 100% identity, including *C. jejuni* 81-176 (GenBank NG_048260.1). Furthermore, our alignment studies using SnapGene2.3.2 demonstrated absolute homology between *tet*(O) (GenBank M18896.2) and Shiraz3 and 4 *tet*(A) sequences (GenBank JX891463.1 and JX891464.1, respectively). We propose that the true identity of the Shiraz 3 and 4 sequences are *Campylobacter tet*(O).

Furthermore, in 2016, a second study reporting a high prevalence of *tet*(A) among thermophilic *Campylobacter* spp. isolated from extensively reared Kenyan broilers was published by Nguyen and coworkers [2]. In that study, the *tet*(A) primers included the same primers as those used by Abdi-Hachesoo et al. [1] (but they produced an anomalous amplicon size) and a second set of in-house designed *tet*(A) primers (designated tet-A-1 and tet-A-2) [2]. However, the primers designed by Nguyen et al. [2] were based on the Shiraz 3 and 4 sequences (GenBank JX891463.1 and JX891464.1, respectively) [1], which we clarified above as *tet*(O). To confirm this, we performed an in silico PCR using SnapGene2.3.2 with the tet-A-1 and tet-A-2 primers [2] and *Campylobacter tet*(O) sequences (GenBank M18896.2 and NG_048260.1). A 486 bp product was predicted, which correlates to the amplicon length reported by Nguyen et al. [2]. We believe that this reported high prevalence of *tet*(A) among this subset (n = 53) of thermophilic *Campylobacter* isolates is erroneous. Our opinion also explains why clusters of *tet*(A) harbouring *Campylobacter* spp. isolates are not described in any database, to our knowledge.

In conclusion, further study would be required to determine whether the homology between *tet*(A) potentially present in *Campylobacter* and known *tet*(A) genes would be sufficient to allow amplification using the primers designed in our study. The investigation of alternative *Campylobacter*-associated tetracycline resistance mechanisms is certainly worthwhile, but the presence of *tet*(A) in *Campylobacter* spp. is an open question.

## Data Availability

The datasets generated and/or analysed during the current study are available in the GenBank repository, https://www.ncbi.nlm.nih.gov/genbank/

## Abbreviations

Bp: base pair
*C. jejuni*: *Campylobacter jejuni*
*C. coli*: *Campylobacter coli*
dNTP: deoxyribonucleoside triphosphates
*E. coli*: *Escherichia coli*
PCR: polymerase chain reaction
spp.: species
